# Variability in the performance of preventive services and in the degree of control of identified health problems: A primary care study protocol

**DOI:** 10.1186/1471-2458-8-281

**Published:** 2008-08-08

**Authors:** Bonaventura Bolíbar, Clara Pareja, M Pilar Astier-Peña, Julio Morán, Teresa Rodríguez-Blanco, Magdalena Rosell-Murphy, Manuel Iglesias, Sebastián Juncosa, Juanjo Mascort, Concepció Violan, Rosa Magallón, Javier Apezteguia

**Affiliations:** 1 Institut d'Investigació en Atenció Primària Jordi Gol (IDIAP Jordi Gol), C/Gran Via de les Corts Catalanes 587 àtic, 08007 Barcelona, Spain; 2 Centro de Salud La Mina, Institut Català de la Salut, C/Mar s/n, 08930 Sant Adrià de Besòs, Barcelona, Spain; 3 Centro de Salud de San Pablo, C/Agudores 7, 50003 Zaragoza, Spain; 4 Dirección de Atención Primaria del Servicio Navarro de Salud, Plaza de la Paz s/n, 6^a ^planta, 31002 Pamplona, Spain; 5 Institut d'Investigació en Atenció Primària Jordi Gol (IDIAP Jordi Gol), C/Gran Via de les Corts Catalanes 587 àtic, 08007 Barcelona, Spain; 6 Centro de Salud El Carmel, Institut Català de la Salut, C/de Murtra, 08032 Barcelona, Spain; 7 UD Centre, Institut Cátalà de la Salut, C/Torrebonica s/n, 08227 Terrassa, Barcelona, Spain; 8 Centro de Salud Florida Sud, Institut Català de la Salut, C/Parc dels Ocellets s/n, 08905 Hospitalet de Llobregat, Barcelona, Spain; 9 Institut d'Investigació en Atenció Primària Jordi Gol (IDIAP Jordi Gol). C/Gran Via de les Corts Catalanes 587 àtic, 08007 Barcelona, Spain; 10 Unidad de Investigación Atención Primaria. Centro de Salud Arrabal. Gracia Gazulla 16. 50015 Zaragoza, Spain; 11 Dirección de Atención Primaria del Servicio Navarro de Salud, Plaza de la Paz s/n 6^a ^planta, 31002 Pamplona, Spain

## Abstract

**Background:**

Preventive activities carried out in primary care have important variability that makes necessary to know which factors have an impact in order to establish future strategies for improvement. The present study has three objectives: 1) To describe the variability in the implementation of 7 preventive services (screening for smoking status, alcohol abuse, hypertension, hypercholesterolemia, obesity, influenza and tetanus immunization) and to determine their related factors; 2) To describe the degree of control of 5 identified health problems (smoking, alcohol abuse, hypertension, hypercholesterolemia and obesity); 3) To calculate intraclass correlation coefficients.

**Design:**

Multi-centered cross-sectional study of a randomised sample of primary health care teams from 3 regions of Spain designed to analyse variability and related factors of 7 selected preventive services in years 2006 and 2007. At the end of 2008, we will perform a cross-sectional study of a cohort of patients attended in 2006 or 2007 to asses the degree of control of 5 identified health problems. All subjects older than16 years assigned to a randomised sample of 22 computerized primary health care teams and attended during the study period are included in each region providing a sample with more than 850.000 subjects. The main outcome measures will be implementation of 7 preventive services and control of 5 identified health problems. Furthermore, there will be 3 levels of data collection: 1) Patient level (age, gender, morbidity, preventive services, attendance); 2) Health-care professional level (professional characteristics, years working at the team, workload); 3) Team level (characteristics, electronic clinical record system). Data will be transferred from electronic clinical records to a central database with prior encryption and dissociation of subject, professional and team identity. Global and regional analysis will be performed including standard analysis for primary health care teams and health-care professional level. Linear and logistic regression multilevel analysis adjusted for individual and cluster variables will also be performed. Variability in the number of preventive services implemented will be calculated with Poisson multilevel models. Team and health-care professional will be considered random effects. Intraclass correlation coefficients, standard error and variance components for the different outcome measures will be calculated.

## Background

The Spanish National Health Service (NHS) provides universal cover and is financed, essentially, by general taxes. The system is divided into primary and secondary care. Primary care (PC) is organized as a network of primary health care teams (PHCT) that behave as geographical and administrative units where PC services are planned, managed and provided for a population ranging from 5,000 to 25,000 citizens. The PHCT staff includes: general practitioners (GPs), paediatricians, nurses, social workers, dentists and ancillary staff. GPs care for individuals older than 14 years (except in rural areas where they look after all members of the local population) and paediatricians look after those between 0 and 14 or 16 years, depending on the region. These clinicians act as gate-keepers for the rest of the public health care system. The PHCT works usually in a single health care centre but in rural areas there may have other small offices.

Secondary care includes out-patient and in-patient hospital care and out-patient care in multi-disciplinary clinics.

The Spanish NHS has been strongly decentralized into 17 Autonomous Communities or regions that configure the Spanish State. Each of these regions has its own governmental structure which, among other responsibilities, provides health-care services to the population. There are minor differences between them with respect to structure and administration. For example, in Aragon and Navarra all services are provided by the State-funded regional service while in Catalonia, the provision of primary care services is offered by different providers (state and non-state funded) among which the Catalan Health Institute (ICS) manages almost 80% of all PHCT. According to latest census of the year 2002, Aragon had a population of 1,209,888 inhabitants while Navarra had 560,235 inhabitants and Catalonia 6,418,387.

### Preventive services in Primary Care

PC is the most accessible health-care level to the general population. In Spain, more than 95% of the population visit their GP at least once in every 5 years. No other health-care level is in a better position to evaluate the global health status of the individual and to decide when to act and what are the ideal measures to take in each specific situation [1].

There is an increasing evidence of the health benefits achieved from the implementation of preventive measures in normal clinical practice. In the last quarter of the century several groups of experts such as the Canadian Task Force on Periodic Health Examination in 1979 [2] and the US Task Force in 1980 [3,4] have published evidence-based recommendations regarding the relevance and the outcomes of the implementation of preventive interventions. Prestigious institutions such as WHO and the Royal College of General Practitioners [5] have highlighted the valuable role of health-care professionals within PCHTs in developing these services.

In Spain, the Spanish Society of Family and Community Medicine *[Sociedad Española de Medicina de Familia y Comunitaria; semFYC] *launched in 1988 the Preventive Activities and Health Promotion Program *[Programa de Actividades Preventivas y de Promoción de la Salud; PAPPS] *that have as main objective promoting the implementation of preventive and health promotion services in PC [6]. The EUROPREV (European Network for Prevention and Health Promotion in Family Medicine and General Practice) was created to extend and coordinate the experiences from this program and to promote preventive services at the European level.

In the period between 1989 and 2003 PAPPS program carried out biennial evaluations of a representative sample of patients attending the PHCTs participating in the program (671 PHCT since the beginning of the program). These evaluations have been the only evaluations of preventive services carried out nation-wide and provide information on the progress and effectiveness of the program over its 15 years of existence [7-9]. From the studies we know that the best-implemented group of preventive services is the minimum package of the adult sub-program which includes screening for hypertension, smoking status and alcohol consumption (done in 92.8% of patients), followed by a second group that includes anti-influenza vaccination, screening for hypercholesterolemia and obesity and anti-tetanus vaccination (done in more than 70% of patients) [8].

However, the progressive implementation of systematic evaluations of primary care performance promoted by the Autonomous health administrations and the progressive computerization of clinical records has brought-about a re-thinking of this evaluation model. The computerization of clinical records provides rich information based on individual data (not centre aggregated data) on a wider and more representative sample that avoids manual and voluntary recording of data. This allows a better study of the clinical practice variability (CPV) and its determining factors.

### Clinical practice variability in preventive services in Primary Care

In Spain, there have been few nation-wide population studies evaluating CPV in PC. This has been due, in part, to the lack of reliable information systems. These studies have often been performed at a hospital level because of the existence of minimum basic data sets (MBDS) provided on discharge from hospital. Nevertheless, from the existing evaluations, PAPPs studies [8,9] and other national [10-13]and international [14-16] studies it is clear that there are important difficulties with respect to the correct implementation of preventive services and health promotion interventions and there is a high variability in their implementation. There is extensive literature on factors related to prevention of specific diseases but less related to implementation of a combination of preventive services as routine clinical practice in PC that may have a profound effect on different diseases. These factors vary considerably, depending on the type of study, the country and type of preventable disease. There are different factors associated with the supply and demand of services that result in elevated variability between and within centres: patient-related factors (socio-demographic and clinical factors), health professional associated factors (specialty, training and professional competence, number of years working with the PHCT), team related factors (existence of specific registries, workload, frequency of doctors encounters, teaching centre) and other health-care provision system characteristics [12].

High CPV translates into problems of clinical effectiveness and social efficiency in health-care provision and, as such, is of concern to health-care providers and to Society in general [17,18]. An analysis of CPV is therefore essential in decision making in the health-care provision politics and prioritization in clinical management (e.g. to generate guidelines for implementation in those areas of high clinical variability).

Hence, it is essential to have studies that include a territory-wide sampling in order to achieve greater representativeness and which take into account methods of design and statistical analysis (e.g. cluster-based analyses) to obtain valid estimates and to explore the influence of patient, health-care professional and PHCT characteristics on the CPV of preventive services in Spain.

### Sources of information in Primary Care: the computerization of clinical records

Primary Care uses electronic clinical records (ECRs) to monitor health problems and to register preventive care services. Although information collection intends to be comprehensive, the ECR systems are different for each region.

Routinely-collected data have undisputed advantages in the study of CPV; they are available almost instantaneously and provide information on a large number of patients. The creation of registries or databases of health-care interest in PC was given a considerable boost with the computerization of clinical records. Spain, contrary to other countries [19-23], has little experience in the use of nation-wide databases containing electronic clinical records in PC. Recently, projects such as the BIFAP project in pharmacoepidemiological research [24] have been initiated in Spain.

However, there are certain difficulties in their use: 1) The data was originally collected for a function different from current research requirements; 2) ECR systems are different in each region, with different standards and computer programs; 3) The heterogeneous degree of implementation in PHCTs; and 4) The degree of exhaustivity of data recorded (for example, some activities are carried out but are not registered). Nevertheless, the potential of this type of registry has been confirmed by different European experiences mentioned earlier [25-27]. As such, the advantages associated to computerization of clinical records are beginning to be appreciated by the different regions and the richness of information provides enormous potential for the study of CPV in primary care.

### Intraclass correlation coefficient

Reports of cluster randomised studies [28] should include sample size calculations and statistical analyses that take clustering into account. The intraclass correlation coefficient (ICC) is a measure of the relatedness of clustered data [29]. Compared with individual randomisation, cluster randomisation may substantially increase the sample size required to maintain adequate statistical power [30].

One of the problems that needs to be faced in designing cluster-based studies (area or organization-based studies) is that estimates of ICC and components of variance for the outcomes of interest need to be obtained from previous studies in order to determine the required sample size for the cluster design [30-32]. There have been repeated calls for publication of ICCs for these types of studies to help others who are planning cluster-based studies [29,33-35].

The differences [30] in ICCs among potential outcome variables, the paucity of appropriate information [36] concerning components of variance or ICC and the lack of data obtained from PC settings reinforce the need for valid estimates that would ensure proper study design.

### Study objectives

The planned objectives in the present study are:

- To describe the variability in the performance of 7 preventive services (detection of smoking status and excessive alcohol consumption; screening for hypertension, obesity and hypercholesterolemia; anti-influenza and anti-tetanus immunization) in a population older than 16 years of age receiving attention in years 2006 or 2007 and to analyse factors related to their implementation.

- To analyse the evolution of 5 identified health problems (decrease in consumption or abstention from tobacco and alcohol and degree of control of hypertension, hypercholesterolemia and obesity) up to December 2008 in the population older than16 years of age attended by the reference PHCT at least once between 2006 and 2007 and followed up during 2008.

- Estimate the ICC, together with the within-cluster and between-cluster components of variance for each of the outcome measures of interest at the PC level.

## Methods/Design

### Design

This is a multi-centered cross-sectional study of a randomised sample of PHCT stratified by region to describe the variability in the implementation of 7 preventive services and their related factors in the period of 2006 or 2007. In 2008, using another cross-sectional study, the evolution or degree of control of 5 of the identified health problems will be assessed, together with the determinants of variability. We use a longer period of study because we need more time to evaluate the impact on the degree of control of these health problems due to changes on life styles.

### Setting

The study is set in PHCTs computerized prior to 1st of January 2005 in the regions of Aragón, Navarra, and Catalonia. So far computerization had occurred in 19% of 121 PHCTs in Aragón, 76% of 54 PHCTs in Navarra, and 97.5% of 278 PHCTs in Catalonia (at the Catalan Health Institute).

### Study population: PHCT and patients

We performed random cluster sampling stratified by region with computerized PHCT used as unit of randomisation.

The inclusion criteria of the PHCT are: 1) computerization of the clinical records before the 1st of January 2005 to ensure familiarity with the system's use; 2) wide use of ECR, and 3) agreement to participate in the study by most of the health-care professionals working in each PHCT (> 80%).

For each computerized PHCT we select the ECRs of all subjects older than16 years as the unit of data collection. For the assessment of variability, the ECR of subjects receiving attention at least once in each of the study years will be extracted from the database. It is estimated that the average number of subjects receiving attention per year is around 70%. Thus in a PHCT in Aragon this figure would be around 10,000 patients, in Navarra around 7,500 and in Catalonia around 12,000. For the analysis of the evolution of health problems and related factors, subjects visited at least on one occasion during the 2006–2007 period and on a further occasion in 2008 will be included.

### Sample size and randomisation

To achieve the equivalent power of a patient-randomised design, in a cluster randomised design the effective sample size will need to be the standard sample size of a single random sampling multiplied by the design effect [31,37,38]. The design effect is a function of the average cluster (computerized PHCT) size and the (intraclass correlation coefficient (ICC) of the outcome:

design effect = 1 + (n-1)*ICC

where *n *is the average number of individuals sampled per cluster [38].

sWith the cluster design we set an ICC of 0.05 on the assumption that the ICCs estimated for outcome variables from PC are less than 0.05 [28,36,39] and that the average number of individuals sampled per centre is at least of 2,500. Therefore 20 computerized PHCTs are necessary from each region to guarantee a statistical power of 80%, assuming a 50% event rate with an alpha two-tailed significance level of 0.05. Finally, to allow any of the randomised computerized PHCTs declining our offer to participate, we increased the number of computerized PHCTs selected from each region to 22. As such, the total number of subjects included will be more than 300,000 in Aragón, 200,000 in Navarra and 350,000 in Catalunya i.e. more than 850,000 subjects in total. This volume of subjects ensures a good estimate of the different outcome measures in the sub-groups of each cluster.

The selection of computerized PHCT to participate in the study is by a simple random sample within each region. Sample size calculation and computerized PHCT selection is performed with Epidat 3.1.

### Outcomes and others measures

#### Primary outcome measures

The **primary outcome measures relating to objective 1 **include the description of the implementation of preventive services according to the PAPPS criteria [6].

Screening for smoking status: if there is information on the consumption of tobacco over the last two years in members of the population who previously were non-smokers.

Screening for excessive alcohol consumption: if there is information on the consumption of alcohol over the last two years in members of the population who previously were not high consumers of alcohol.

Screening for obesity: if there have been measurements of height and weight or body mass index (BMI) over the last 4 years in members of the population who previously were non-obese.

Screening for hypertension: if there is information on systolic blood pressure (BPS) and diastolic blood pressure (BPD) during the last 4 years in members of the population aged between 16 and 40 years who previously did not have the diagnosis of hypertension; or over the past 2 years in members of the population older than 40 years who previously did not have the diagnosis of hypertension.

Screening for hypercholesterolemia: if there is information on total blood cholesterol corresponding to the following criteria of age and gender: 1 measurement in males between 16–35 years old; 1 measurement in females between 16–45 years old; 1 measurement every 5 years in males between 35–75 years old; 1 measurement every 5 years in females between 45–75 years old; 1 measurement if there has not been a previous measurement in those older than 75; all of these performed in population that previously did not have the diagnosis of hypercholesterolemia.

Anti-tetanus vaccination (ATV): if there is a registry of anti-tetanus vaccination over the previous 10 years.

Anti-influenza vaccination (AIV): if there is a registry of annual anti-influenza vaccination in those older than 59 years of age.

The **primary outcome measures relating to objective 2 **describes the clinical follow-up of the five health problems identified up to December 31^st ^2007 and evaluated up to December 31^st ^2008:

Evolution of tobacco consumption: quit smoking or remaining an ex-smoker.

Evolution of excessive alcohol consumption: cessation of consumption or decrease below the definition of excessive consumption (28 alcohol units/week in males and 17 alcohol units/week in females, where 1 alcohol unit = 10 grams of alcohol).

Evolution of obesity: decrease in BMI to <30 kg/m^2 ^or change in the diagnosis to non-obese.

Evolution of hypertension: mean of the last 2 determinations of systolic BP/diastolic BP registered as being ≤ 130/80 mmHg in patients of high risk (diagnosed with diabetes, renal or cardiac insufficiency) and ≤ 140/90 mmHg in the rest of the population.

Evolution of hypercholesterolemia: last measurement of LDL cholesterol registered as being ≤ 100 mg/dL in high-risk patients (diagnosed with diabetes, ischaemic heart disease or cerebrovascular accident) and ≤ 130 mg/dL in the rest of the population.

#### Secondary outcome measures

Number of different preventive services (among the 7 of the study) registered for each individual during the 2006 and 2007 year periods.

#### Other measures

We assess the following factors related to the outcome measures according to the levels of analysis:

Computerized PHCT: variables of structure and organization of the PHCT (number of GPs and nurses, presence of specialists), adherence to PAPPS, training centre for family doctors, ECR system and starting year, assigned population.

Health-care professionals (GPs, nurses): gender, age, specialty, years of employment in PC and in the PHCT, workload (mean number of visits/day), years of experience with ECR.

Subjects: gender, age, comorbidity according to the Adjusted Clinical Groups grouper [40,41], frequency of consultations (visits/year).

#### Information source

The individual data will be extracted directly from the central computer servers of the ECRs of the subjects included in the study. In Navarra and Aragón the system used is OMI-AP and in Catalonia the eCAP system. The data related to health-care professionals and to PHCT will be obtained from an on-line database for health-care professionals and from a person responsible in each centre.

#### Data extraction and processing

Using the specific interface for each specific computer system (OMI-AP and eCAP), each region will extract the study data and transfer them to a central database where they will be homogenized and processed into a specially-constructed final database.

#### Confidentiality of data

Data confidentiality will be assured during and subsequent to the extraction using encryption and dissociation of the individual's identification, health-care professionals, and of PHCTs.

#### Ethical aspects

The study has been favourably evaluated by the Research Committee of the IDIAP Jordi Gol.

#### Quality control

Quality controls will be carried out in the 2 extractions of data comparing agreement between the central database and the original data of some centres as for: number of patient, visits and diagnoses as well as data of prevalence of certain diseases. It will also be checked if the population data is appropriate by comparing it with data from the census and other official sources. In the case of items related to clinical activities (for example blood pressure measurement), any register with missing values will be considered as not performed.

### Statistical analyses

For the first two objectives all the analyses will be performed on a global level and each region separately. Cluster level analysis (computerized PHCT and health-care professional) will be performed using standard parametric or non-parametric analytical methods, with cluster means or proportions as observations [37,42]. For the adjusted analysis we will perform regression analysis of individual level data using methods for clustered data, multilevel, hierarchical or random effects models [37,43]. This methodology facilitates simultaneous analyses of the effects of individual and cluster variables in order to evaluate whether the relationship between variables at the individual level varies in accordance with the characteristics of the cluster and whether the individual and cluster variability can be attributed to factors of the cluster and/or to the factors related to the individual. Analysis will be performed at 3 levels: PHCT, health-care professional and individual subjects.

Multilevel linear (for continuous outcome variables) or logistic (for dichotomous outcome variables) regression analyses will be performed for the first two objectives. The analyses will be adjusted for explanatory factors at individual, health-care professional and PHCT level and for other variables that are clinically relevant. We will also adjust for possible confounders checking whether they will affect the outcomes. A nested or hierarchical structure will be considered: the attended individuals are nested within the health-care professional and the health-care professional within PHCT. The PHCT and the health-care professional will be considered random effects.

Multilevel Poisson regression models will be used to evaluate the number of preventive activities recorded for each individual.

For the third objective, the ICCs, their standard errors and the components of variance [36,37,42,43] for the different outcome measures will be calculated using methods that enable adjustment for covariables, together with random effect methods (health-care professionals and PHCT). Initially, we will estimate the coefficients of variance and ICCs using the outcome as the dependent variable and the health-care professional/PHCT as random effects. Subsequently, we will perform an adjusted analysis for the characteristics of the individual, the health-care professional and the PHCT.

We will study interactions and collinearity. The collinearity of the maximal models will be evaluated using the criteria proposed by Belsley [44]. We will set the significance level at 1% (two-tailed).

Analysis will be carried out using the SPSS statistical package for Windows, version 15 (SPSS Inc., Chicago, IL) and the SAS package 9.1.3 for Windows (SAS Institute Inc., Cary, NC, USA).

## Discussion

The project results will provide very useful information on the degree of registration and performance of the 7 preventive services selected. These 7 activities have been widely implemented and have a high impact on the health of our population [2-6]. The large population database of the study, with more than 850,000 people recorded, will increase considerably the external validity of the results and will reflect usual clinical practice at the Primary Care settings in the Spanish NHS.

This fact may help to overcome the current limitations and to investigate CPV and the factors that have an impact on the implementation of preventive services.

Certainly the source of information used and the ECRs may have limitations. The heterogeneity of current computer systems may complicate the extraction of outcomes and of variables. However, the design of the data extraction interfaces and data transfer will minimize the problems. The lack of exhaustivity in data recording may introduce some distortions in the information. Nevertheless, stability of computer systems at the centres will help to minimize this problem and the quality control measures will help to identify the magnitude of the problem.

Studies based on cluster sampling allow important reduction in costs and greater easiness in administration but require knowledge of the ICCs. For this study ICCs has been estimated from studies conducted in other countries. In our study we will provide information concerning the magnitude of the ICC, evaluate factors that influence their magnitude, estimate within-cluster and between-cluster components of variance for the outcome measures under consideration and determine the sample size; all of these factors will aid the design and statistical analysis of future cluster-based studies set in PC.

In conclusion, the present study will provide new knowledge in:

- The identification of factors related to the variability in the implementation of preventive services, which will facilitate the design of strategies for improvement in the planning and administration of services in PC.

- The assessment of the evolution of the detected health problems will improve the degree of existent evidence taking into account the results of screening and the control of those problems. This will be very useful for the evaluation of the PAPPS and other studies based on this source of information.

- The estimation of the ICC will be of considerable help in the design, calculation of sample size and analysis of future studies based on the randomisation of clusters in our environment.

- Finally, although not being an aim of the project, information will be obtained on the characteristics of the different computer systems and this information will generate recommendations for the standardization of systems for future applications.

## Competing interests

The authors declare that they have no competing interests.

## Authors' contributions

BB and CP are the principal investigators responsible for the conception of the project and drafting the manuscript. TR was in charge of the design of sample calculation and statistical analyses.  MPA, JM, MR, CV, MI, RM, SJ, JMP, and JA contributed to the description of the background, general design and definition of the different study variables and their adaptation to the different computerized clinical records systems.

All authors have read and approved the final manuscript

## Pre-publication history

The pre-publication history for this paper can be accessed here:


